# Stringent Response Regulates the Persister Formation and Virulence of *Vibrio splendidus*

**DOI:** 10.3390/microorganisms14061278

**Published:** 2026-06-05

**Authors:** Meishan Qin, Yuehui He, Yuanyuan Zhou, Peng Zhang, Chenghua Li, Shanshan Zhang

**Affiliations:** 1 State Key Laboratory for Managing Biotic and Chemical Threats to the Quality and Safety of Agro-Products, Ningbo University, Ningbo 315211, China; qms0905@163.com (M.Q.); lichenghua@nbu.edu.cn (C.L.); 2 Single-Cell Center, CAS Key Laboratory of Biofuels, Shandong Key Laboratory of Energy Genetics, Qingdao Institute of Bioenergy and Bioprocess Technology, Chinese Academy of Sciences, Qingdao 266101, China; heyh@qibebt.ac.cn (Y.H.); zhouyy@qibebt.ac.cn (Y.Z.); 3 College of Materials Science and Engineering, Qingdao University, Qingdao 266071, China; zhangpeng1@qdu.edu.cn; 4 Laboratory for Marine Fisheries Science and Food Production Processes, Qingdao National Laboratory for Marine Science and Technology, Qingdao 266071, China

**Keywords:** stringent response, single-cell Raman spectroscopy, *Vibrio splendidus*, persister

## Abstract

*Vibrio splendidus* is an important opportunistic pathogen that causes diseases in aquatic animals, and its persisters increase the difficulty of aquaculture disease control. The stringent response is a central pathway in bacteria for coping with environmental stress, and the signaling molecule (p)ppGpp, synthesized under the regulation of RelA/SpoT homologs, is closely associated with persister formation and virulence modulation. However, the regulatory mechanisms linking the stringent response to persister formation and virulence in *V. splendidus* remain unclear. In this study, the core gene deletion strains Δ*relA* and Δ*relA*Δ*spoT* were constructed via homologous recombination. Combined with D_2_O single-cell Raman spectroscopy, transcriptomics, and phenotypic assays, we systematically characterized the biological effects of stringent response inactivation. The results showed that the loss of *relA* and *spoT* significantly reduced persister formation and key virulence traits while enhancing biofilm formation. Single-cell Raman spectroscopy analysis indicated that persisters remained metabolically active, accompanied by changes in different cellular components. Transcriptome analysis revealed that the absence of stringent response affected multiple pathways, including ribosomal function, energy metabolism, two-component systems, and quorum sensing. Additionally, the sigma factor RpoS may potentially exert a compensatory function in Δ*relA*Δ*spoT* strain, but this requires further validation. In conclusion, the stringent response positively regulates persister formation and virulence in *V. splendidus*, despite the existence of complex regulatory mechanisms. This study provides a theoretical basis for the development of anti-infective strategies targeting stringent response in aquatic pathogens.

## 1. Introduction

Bacteria frequently encounter adverse environmental factors during growth, and rapid sensing and adaptation are required for their survival [1]. The stringent response is a conserved stress pathway enabling rapid environmental adaptation [2]. This response is characterized by the accumulation of (p)ppGpp, nucleotides synthesized and degraded by the RelA/SpoT homolog (RSH) protein superfamily and formed from GDP and GTP [3]. RSH enzymes vary among species and are classified into multi-domain long enzymes and single-domain short enzymes [4]. Most Proteobacteria possess two long RSH enzymes. RelA exhibits only synthetic activity due to a mutated hydrolysis domain, and SpoT is bifunctional but possesses relatively weak synthetic activity [5]. The stringent response coordinates transcription and replication under stress and is implicated in virulence and persister formation.

The link between the stringent response and persister formation has attracted much attention. Persisters are stress-induced bacterial subpopulations that tolerate environmental pressures through transient growth arrest without genetic changes [6]. They resume growth after stress removal while retaining stress sensitivity [7,8]. In *Escherichia coli*, high-frequency persister formation in the *hipA7* strain depends on both the *relA* and *spoT* genes. Deletion of *relA* attenuates this phenotype, and double deletion abolishes (p)ppGpp synthesis and the high-persistence phenotype [9]. (p)ppGpp accumulation induced by tRNA starvation increases *E. coli* persister levels [10], and the PRDP model suggests ppGpp induces persisters by regulating ribosome activity [11]. In addition, persister formation by *Salmonella* within macrophages was found to be associated with the ppGpp/Lon protease-dependent TA system [12]. In *Pseudomonas aeruginosa*, inactivation of the stringent response by disruption of the *relA* and *spoT* genes significantly reduces antibiotic tolerance [13]. The stringent response is also associated with bacterial virulence. (p)ppGpp deficiency has been shown to attenuate pathogen virulence and significantly impair pathogenicity [14]. This is because these mutants exhibit metabolic disorders and heightened stress sensitivity, impairing host adaptation and colonization [15]. Furthermore, (p)ppGpp directly or indirectly controls the expression of genes involved in AHL and PQS quorum-sensing systems and also promotes biofilm formation in *P. aeruginosa* [16]. The stringent response is critical for persister formation, antibiotic tolerance, and virulence in many pathogens [2,5]. Loss of this function markedly affects bacterial stress adaptation and survival capacity. However, investigations into the mechanisms by which the stringent response governs persister formation and virulence regulation in aquatic pathogens remain limited.

*Vibrio splendidus* is a widespread marine opportunistic pathogen and the primary cause of skin ulceration syndrome in *Apostichopus japonicus*. [17]. Previous work from our laboratory has confirmed that this bacterium can induce persister formation under antibiotic pressure and host immune factors [18]. Furthermore, the RT-PCR results suggested that the stringent response may regulate persister formation. Persisters complicate disease control and promote excessive antibiotic use, leading to microecological imbalance, drug residues, and resistance spread [19]. Consequently, an in-depth elucidation of the metabolic characteristics and formation mechanisms of *V. splendidus* persisters has important theoretical value and urgent practical significance for breaking the bottleneck of aquaculture disease control.

In fact, in-depth studies of persisters have long faced significant technical challenges. Traditionally, persister identification has relied on population-level antibiotic killing curves [20]. However, the true physiological status of persisters at the single-cell level remains poorly understood. Within this context, single-cell Raman spectroscopy (SCRS) exhibits unique advantages [21]. Raman spectra provide molecular fingerprints of chemical composition, enabling the identification of functional changes in cells and tissues [22]. Biochemical constituents, including information on nucleic acids, proteins, lipids, and carbohydrates are reflected by these spectra [21,23]. When combined with deuterium (D) isotope labeling, single-cell metabolic activity can be precisely quantified [24]. The principle is that metabolically active cells growing in D_2_O-containing medium incorporate deuterium into biomacromolecules, forming C-D bonds that replace C-H bonds. A characteristic C-D peak appears in the 2050–2300 cm^−1^ region of the Raman spectrum. The intensity of this signal is proportional to the amount of deuterium incorporation, enabling non-destructive quantitative characterization of single-cell metabolic activity [25]. This D_2_O-labeled SCRS has been successfully applied in studies of persister cells in *Mycobacterium tuberculosis* [26] and *E. coli* [27]. It provides a powerful methodological framework for investigating *V. splendidus* persisters under in situ and non-destructive conditions. Based on this, core gene deletion strains of the *V. splendidus* stringent response (Δ*relA* and Δ*relA*Δ*spoT*) were constructed in this study via homologous recombination. D_2_O-SCRS and transcriptomics were employed to analyze the regulatory effects of the stringent response on persister formation, antibiotic susceptibility, and growth- and virulence-associated phenotypes. The findings are expected to further elucidate the molecular mechanisms by which the stringent response mediates persister formation and virulence expression in *V. splendidus*. This study will provide experimental evidence and a theoretical foundation for the development of anti-infective strategies targeting the stringent response.

## 2. Materials and Methods

### 2.1. Bacterial Strains

The *Vibrio splendidus* strain AJ01 was isolated, identified and preserved in our laboratory; its derivatives Δ*relA* and Δ*relA*Δ*spoT* were obtained in this study. Specific information is provided in Table 1 below.

### 2.2. Experimental Animals

Healthy *Apostichopus japonicus* juveniles (weight: 3.2 ± 1.1 g, length: 2.2–3.6 cm) used in this study were purchased from a commercial aquaculture farm in Dalian, China. The juveniles were acclimated for 7 days in a recirculating seawater system with moderate aeration at a salinity of approximately 28 ppt and a temperature of 16 °C. Subsequent stress experiments were performed after they had stably adapted to the environment.

### 2.3. Growth Conditions and Antibiotics

The culture media used in this study were LB (10 g/L tryptone, 5g/L yeast extract, and 10 g/L NaCl) for the culture of *Escherichia coli*, 2216E (10 g/L tryptone, 5 g/L yeast extract, and 35 g/L NaCl), and LB_35_ (10 g/L tryptone, 5 g/L yeast extract, and 35 g/L NaCl). Both 2216E and LB_35_ media were used for the culture of *V. splendidus*. Antibiotics and other reagents were prepared in sterile ultrapure water if not otherwise specified. Commonly used antibiotics included ampicillin (100 mg/mL), kanamycin (50 mg/mL), ciprofloxacin (10 mg/mL), streptomycin (100 mg/mL), gentamicin (10 mg/mL), and tetracycline (10 mg/mL).

### 2.4. Construction of Mutant Strains

In-frame deletion mutants of *relA* and *spoT* were constructed via *sacB*-mediated allelic exchange using the suicide vector pK18mobsacB. Based on the complete genome sequence of *Vibrio splendidus* AJ01, which was sequenced and assembled by our laboratory (unpublished), the knockout primers were designed according to the overlap extension principle. Then, 600 bp upstream and downstream homologous fragments of *relA* and *spoT* were amplified with primer pairs KoRelAF1/R1, KoRelAF2/R2, KoSpoTF1/R1, and KoSpoTF2/R2, respectively. The obtained fragments were assembled into the digested pK18mobsacB vector with a seamless cloning kit, followed by transformation into DH5α competent cells. Verified recombinant plasmids were transformed into *E. coli* S17λπ. Through bacterial conjugation, the recombinant plasmid pK18-KoRelA was transferred into the *V. splendidus* AJ01 strain. The first round of homologous recombination was selected on agar plates containing ampicillin and kanamycin. Positive clones were identified by PCR using the primer pair KoRelAF1/R2. The second round of homologous recombination was performed on agar plates containing 12% (*w*/*v*) sucrose. Finally, the Δ*relA* mutant was confirmed by PCR and sequencing. The Δ*relA*Δ*spoT* double mutant strain was constructed by transferring the recombinant plasmid pK18-KoSpoT into the Δ*relA* strain and undergoing two rounds of homologous recombination. The primers required for the construction of the mutant strains are listed in Table 2.

### 2.5. Antibiotic-Induced Persisters

As mentioned earlier [18], persister cell formation in *V. splendidus* was investigated by exposing the bacteria to tetracycline. Overnight cultures of *V. splendidus* were inoculated into fresh LB_35_ medium and incubated with shaking at 28 °C until an OD_600_ of 1.0 was achieved. To induce persister formation, 50 μg/mL tetracycline (~32× MIC) was added to the culture, and the bacteria were treated for 4 h at 28 °C to kill non-persisters. Subsequently, 1 mL of the bacterial culture was harvested, washed three times with sterile PBS, and serially diluted with PBS. Then, 10 μL of the diluted samples were spotted onto LB_35_ agar plates and incubated overnight at 28 °C. Colony-forming units were counted to evaluate bacterial persistence.

### 2.6. Single-Cell Raman Spectroscopy

The overnight culture of *V. splendidus* was inoculated into fresh LB_35_ medium and incubated at 28 °C with shaking until the OD_600_ reached 1.0. Subsequently, tetracycline was added, and the suspension was incubated for 4 h to induce persister formation. Bacterial cells were pelleted by centrifugation and resuspended in medium containing 25% D_2_O, followed by incubation at 28 °C for 6 h for isotopic labeling. During this period, tetracycline was continuously added to maintain the persister state. Bacterial cells were then harvested by centrifugation and washed three times with Milli-Q water to remove potential contaminants that could interfere with Raman spectroscopy detection. The resulting bacterial pellet was resuspended in ultrapure water to disperse the cell aggregates into single cells. A 1.5 μL bacterial suspension was spotted onto CaF_2_ slides and allowed to air dry at room temperature. The single-cell Raman spectra were acquired using a WiRE 5.3 (Renishaw, Wotton-under-Edge, UK) confocal micro-Raman system with a 532 nm Nd:YAG excitation laser and a 900 grooves/mm diffraction grating. A 100× dry objective with a numerical aperture of 0.9 (Olympus, Tokyo, Japan) was utilized to observe bacterial cells and collect Raman signals. The laser power on the sample was approximately 30 mW/µm^2^. Raman spectra were recorded between 450 and 3100 cm^−1^, with a spectral resolution of about 2 cm^−1^. Each spectrum was acquired over a period of 5 s, and at least 100 valid single-cell spectra were collected per sample, adhering to quality control protocols. Quality control involved excluding spectra that displayed background signals, impurity signals, or noticeable fluorescence signals. After baseline correction and intensity summation normalization pretreatment of the Raman spectrum, the data were visualized using Origin 2021. The individual spectral bands were assigned according to their physical origin and vibrational modes in the cellular compounds (Table 3).

### 2.7. Determination of Bacterial Growth Curves

*V. splendidus* was inoculated into medium and cultured overnight in a shaking incubator at 28 °C. The bacterial suspension was then diluted 1:1000 into fresh 2216E medium, and 200 µL of the mixture was added to a 96-well plate with 5–6 replicate wells per group. The plate was incubated at 28 °C with shaking for 48 h in the Tecan Spark microplate reader. The detection program was initialized with the initial time set to zero, and the absorbance at 600 nm (OD_600_) was automatically recorded hourly. Finally, the bacterial growth curve was plotted against incubation time and corresponding OD_600_ values.

### 2.8. Experimental Infection

Healthy *Apostichopus japonicus* juveniles were placed in a 100 L water tank for acclimatization for 3–5 days at a temperature of 16 °C, with daily seawater changes. Subsequently, the *A. japonicus* were randomly distributed into small rearing tanks, with 30 individuals per tank. The experiment consisted of four groups, each with three replicates. The first group served as the negative control untreated with bacteria. The others were infected with *V. splendidus* wild-type (WT), mutant strain Δ*relA* and Δ*relA*Δ*spoT*, respectively. The final bacterial concentration was 1.0 × 10^7^ CFU/mL. The *A. japonicus* were monitored every 12 h, and those that detached from the container wall or exhibited epidermal rupture were considered to have died. Survival curves were generated using GraphPad Prism 8.0.2 (Kaplan–Meier method), and differences between groups were assessed by the log-rank (Mantel–Cox) test. A *p*-value < 0.05 was considered statistically significant.

### 2.9. Bacterial Invasion Experiment

The ability of wild-type and mutant strains to invade was evaluated using the coelomocytes of *A. japonicus*. Healthy *A. japonicus* were selected and dissected from the oral region using sterilized scissors to collect coelomic fluid. Tissue debris was filtered through a 200-mesh sieve, followed by the addition of an equal volume of isotonic anticoagulant (0.48 M NaCl, 0.019 M KCl, 0.02 M EGTA, 0.068 M Tris-HCl, pH 7.4). The mixture was centrifuged at 800× *g* for 10 min at 4 °C to harvest the cell pellet. The pellet was washed three times with isotonic buffer (0.53 M NaCl, 0.001 M EGTA, 0.01 M Tris-HCl, pH 7.6). Coelomocytes were gently resuspended in L-15 medium (0.39 M NaCl, 100 μg/mL Gm, 100 U/mL dual antibiotics). A 2 mL aliquot of the cell suspension was added to each well of a 6-well plate, mixed well, and incubated at 16 °C overnight until the cells adhered. The supernatant medium was carefully removed, and wells were washed three times with PBS to eliminate residual antibiotics and extracellular dead bacteria. Fresh antibiotic-free L-15 medium was subsequently added. Bacterial suspension of *V. splendidus* with an OD_600_ of 1.0 was inoculated into each well, and co-cultivation with coelomocytes was performed at 16 °C for 2 h. After incubation, the supernatant was discarded, and wells were washed three times with PBS to remove non-invasive bacteria. Subsequently, the cells were resuspended in 1 mL of PBS and lysed by repeated pipetting, followed by 10-fold serial dilution. A 10 μL aliquot from each diluted sample was plated on agar plates, and colony counts were performed after incubation at 28 °C for 24 h.

### 2.10. Minimum Inhibitory Concentration (MIC) Assay

The MIC of antibiotics against the wild-type and mutant strains of *V. splendidus* was determined. Briefly, overnight bacterial cultures were diluted 1:1000 into 2216E medium containing a twofold serial dilution of antibiotics and the mixtures were aliquoted into 96-well microplates. The plates were incubated at 28 °C. Absorbance at 600 nm (OD_600_) was measured via a microplate reader at initial inoculation and after overnight incubation. The negative control group consisted of sterile medium without bacterial inoculation was set as the negative control. The MIC value was defined as the lowest antibiotic concentration at which visible bacterial growth was completely inhibited.

### 2.11. Swimming Motility Assays

Bacterial swimming motility was assessed using 0.2% soft agar medium. Overnight cultures of *V. splendidus* were inoculated into fresh medium and incubated with shaking at 28 °C until an OD_600_ of 1.0 was achieved. The bacterial suspension was then diluted 1:100, and 5 μL of the diluted culture was spotted onto the center of soft agar plates. Plates were incubated at 28 °C for 24 h, and the diameter of the motility halo was subsequently measured. The experiment was performed in triplicate, with six replicates per group.

### 2.12. Biofilm Formation

Biofilm formation capacity of bacteria was determined by crystal violet (CV) staining assay [35]. Overnight cultures of *V. splendidus* were diluted 1:1000 into 2216E medium and added into 96-well polystyrene plates, followed by static incubation at 28 °C for 48 h. After incubation, wells were gently washed twice with PBS to remove non-adherent bacteria. Biofilms were stained with 1% crystal violet solution at room temperature for 15 min, then thoroughly rinsed with sterile water to eliminate unbound dye. Subsequently, 200 µL of 95% ethanol was added to each well to solubilize the dye bound to biofilm biomass and incubated for 30 min. Absorbance was measured at 570 nm using a microplate reader and normalized to the OD_600_ of the culture to quantify biomass. The experiment was performed in triplicate, with six replicates for each independent experiment.

### 2.13. Protease Activity Assays

Bacterial extracellular protease activity was determined using a previously described method with minor modifications [36]. Briefly, overnight cultures of *V. splendidus* were diluted 1:1000 into 2216E medium and incubated with shaking at 28 °C. Samples were collected at 0 h, 6 h, 12 h, 24 h, 36 h, and 48 h. Following OD_600_ measurement, the remaining bacterial suspensions were preserved at −20 °C. Samples were thawed on ice and vortexed thoroughly. A 500 µL aliquot of the bacterial suspension was transferred into 1.5 mL centrifuge tubes and centrifuged at 10,000 rpm for 10 min at 4 °C. The supernatant was collected and filtered through a 0.22 µm filter membrane to obtain cell-free sterile supernatant. For each group, 50 µL of the supernatant was mixed with 450 µL azo-casein solution in a new tube, followed by incubation in a 37 °C bath for 2 h. After incubation, 500 µL of 10% trichloroacetic acid was added to terminate the reaction, and the mixture was incubated on ice for 15 min. Then samples were centrifuged at 5000 rpm for 2−5 min. The supernatant was added into a 96-well polystyrene plate with three replicate wells per sample, and the absorbance at 350 nm was measured using a microplate reader. The relative protease activity was expressed as the ratio of A_350_/OD_600_.

### 2.14. Hemolytic Activity Assays

Bacterial hemolytic activity was determined as described below. Overnight cultures of *V. splendidus* were inoculated into 2216E medium and incubated with shaking at 28 °C until an OD_600_ of 1.0 was achieved. The bacterial cultures were then diluted 1:100, and 5 μL of the dilution was spotted onto the center of blood agar plates supplemented with 5% defibrinated sheep blood. The plates were incubated at 28 °C for 48 h. Hemolytic zones were observed under transmitted light, and their diameters were measured. The experiment was performed in triplicate, with six technical replicates for each independent experiment.

### 2.15. Transcriptome Sequencing

Overnight cultures of the wild-type and Δ*relA*Δ*spoT* double mutant strains were inoculated at a ratio of 1:1000 into LB_35_ medium and cultured with shaking at 28 °C until an OD_600_ of 1.0 achieved. Tetracycline was added, and the bacterial cultures were incubated for 4 h to prepare persister samples, with untreated cultures serving as control groups. RNA extraction, cDNA library preparation, Illumina sequencing, and read mapping were performed by Shanghai Majorbio Bio-Pharm Technology Co., Ltd. (Shanghai, China). Three independent biological replicates were performed for each experimental group. Raw sequencing data were subjected to quality trimming and filtering to obtain clean reads. These reads were aligned to the *V. splendidus* reference genome ASM2281204 v1 (accession number: GCF_022812045.1) using the Burrows-Wheeler algorithm. Genes were functionally annotated against the NR, Swiss-Prot, Pfam, COG, GO and KEGG databases. Gene expression levels were quantified using RSEM 1.3.3 with TPM as the indicator. Biological reproducibility was verified by principal component analysis (PCA) and sample correlation analysis. Differentially expressed genes (DEGs) were quantified via DESeq2 1.42.0. Genes with an adjusted *p*-value (Padj) < 0.05 and |log2 fold change| ≥ 1 were defined as showing significant differential expression. GO functional enrichment and KEGG pathway enrichment analyses were conducted using Goatools 1.4.4 and R 4.5.1, respectively. An adjusted *p*-value (Padj) < 0.05 was used as the criterion for significant enrichment of DEG functions and pathways. The raw RNA-seq data have been deposited in the NCBI Sequence Read Archive (SRA) database under the accession number PRJNA1470522.

## 3. Results

### 3.1. Construction and Verification of Mutant Strains

**Figure 1:**
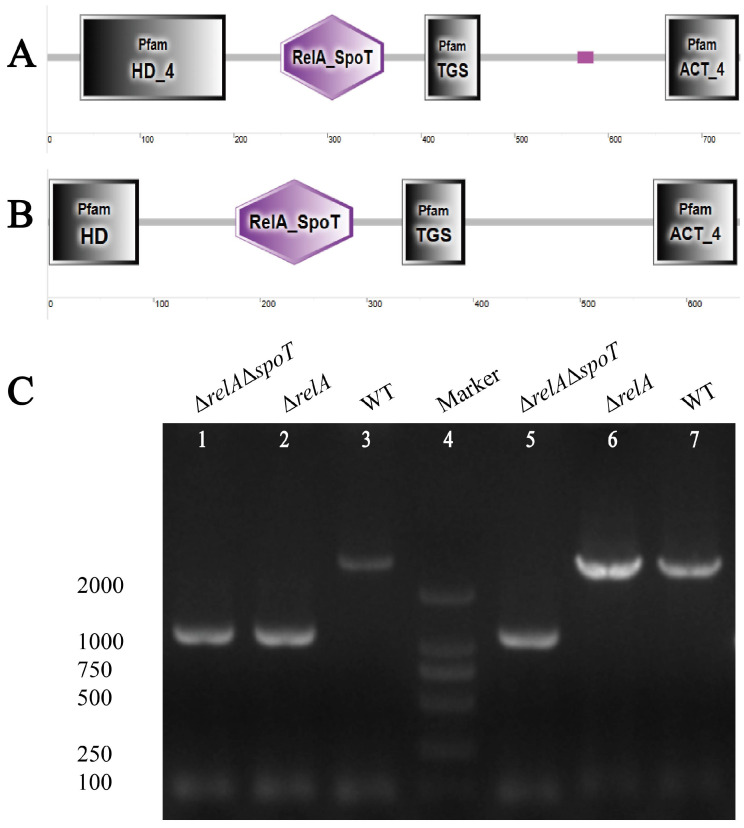
(**A**) Protein domains of RelA in *V. splendidus*, annotated by SMART (https://smart.embl.de/, accessed on 1 July 2025). (**B**) Protein domains of SpoT in *V. splendidus*, annotated by SMART. (**C**) PCR verification results of defective strains. Lanes 1, 2 and 3 show the PCR verification of *relA* gene, and lanes 5, 6 and 7 show the verification of *spoT* gene.

To explore the functions of the stringent response in persister formation and virulence regulation in *V. splendidus*, protein domain analysis was first performed on its core genes *relA* and *spoT*. Prediction via the SMART online tool indicated that the two proteins share highly similar architectures. Both proteins possess a RelA_SpoT synthetase domain, an HD hydrolase domain, a TGS domain, and an ACT_4 domain (Figure 1A,B). The difference is that RelA contains an inactive HD_4 hydrolase domain, and it harbors an additional signal peptide spanning amino acid residues 550–600 compared with SpoT. Such structural divergence is speculated to result in functional differentiation of the two proteins in (p)ppGpp synthesis and hydrolysis. Accordingly, in-frame deletions of the *relA* and *spoT* genes were constructed by homologous recombination, including the single mutant Δ*relA* and the double mutant Δ*relA*Δ*spoT*. PCR verification was carried out using specific primer pairs KoRelAF1/R2 and KoSpoTF1/R2, and the lengths of PCR-amplified fragments were compared among wild-type (WT) and mutant strains. The *relA* fragment was 3330 bp in the WT strain and was shortened to 1104 bp in both the Δ*relA* and Δ*relA*Δ*spoT* strains (Figure 1C, lanes 1–3). For the *spoT* gene, the amplified fragment was 3192 bp in WT strain, and reduced to 10strain and Δ*relA*Δ*spoT* strain (lanes 5 and 7). Meanwhile, sequencing and alignment of the PCR products confirmed successful in-frame deletion of the *relA* and *spoT* genes, and confirmed the construction of Δ*relA* and Δ*relA*Δ*spoT* mutant strains.

### 3.2. Effect of Gene Deletion on Persister Formation in V. splendidus

**Figure 2:**
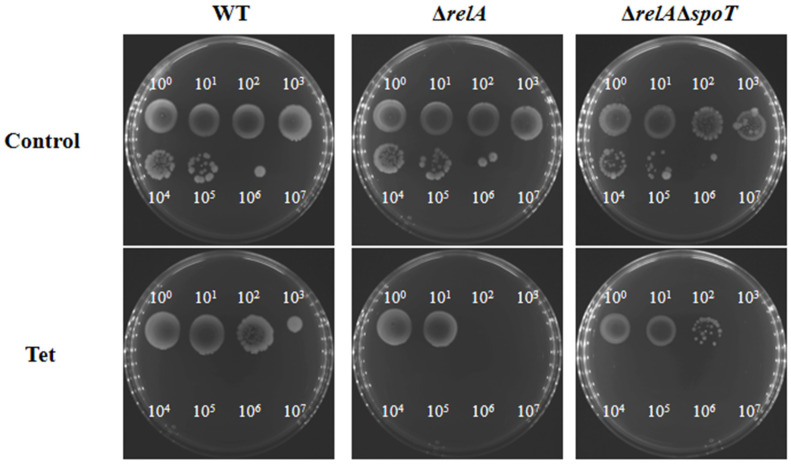
Persister formation of WT, Δ*relA* and Δ*relA*Δ*spoT* strains following tetracycline induction. Serial dilution plate counting was conducted after 4 h of tetracycline treatment. Control group was not exposed to tetracycline.

As previously described [18], persister formation in WT and mutant strains was induced by exposure to 50 μg/mL tetracycline. Under control conditions without tetracycline treatment, no significant difference in viable cell counts was observed among the WT and mutant strains. After tetracycline treatment, abundant persisters were generated in the WT strain. The persister level of Δ*relA* was significantly lower than that of WT. Although the persister quantity of Δ*relA*Δ*spoT* remained lower relative to WT, it displayed partial recovery compared with the Δ*relA* (Figure 2). These results indicate that *relA* and *spoT* jointly regulate persister formation in *V. splendidus*.

**Figure 3:**
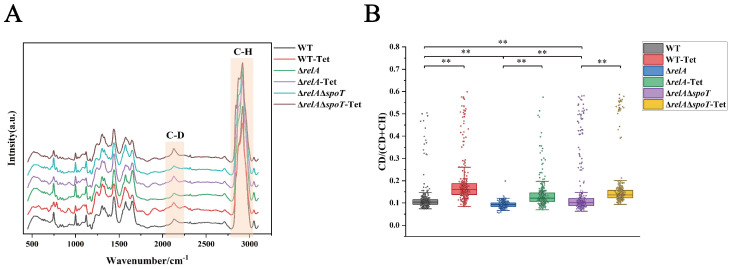
Metabolic activity of WT, Δ*relA*, Δ*relA*Δ*spoT* and their persisters. (**A**) Mean spectra of *V. splendidus* and its persisters, normalized by the sum of fingerprint area from 450 to 3100 cm^−1^. (**B**) CD Ratio of *V. splendidus* and its persisters. N > 100 for each group. ** *p* < 0.01.

Metabolic activity of *V. splendidus* persisters was assessed by D_2_O-labeled Raman spectroscopy [27], with the deuterium incorporation rate [CDR = CD/(CD + CH)] serving as the quantitative indicator. Wild-type and mutant strains were exposed to tetracycline for 4 h to induce persister formation. Subsequently, cells were incubated in medium containing 25% D_2_O and tetracycline for 6 h, followed by collection and cellular Raman spectrum analysis. In Raman spectra, a broad peak ranging from 2050 to 2300 cm^−1^ corresponds to C-D bond formation. The intensity of the C-D peak is positively correlated with both D_2_O concentration and incubation duration, and shows a linear relationship with intracellular metabolic activity. Single-cell Raman spectra of the WT, Δ*relA* and Δ*relA*Δ*spoT* strains and their corresponding persisters were detected. The results showed that all three strains exhibited active metabolism after tetracycline treatment (Figure 3B). The basal metabolic activity of the Δ*relA* was lower than that of the WT strain. Following persister induction, its metabolic activity was upregulated but remained lower than that of WT persisters. In contrast, the basal metabolic activity of the Δ*relA*Δ*spoT* was higher than that of the WT. Its persister metabolic level exhibited partial recovery compared with the Δ*relA* single mutant, but was still lower than that of the WT persisters. These findings suggest that the *relA* and *spoT* genes may influence bacterial anabolic metabolism.

**Figure 4:**
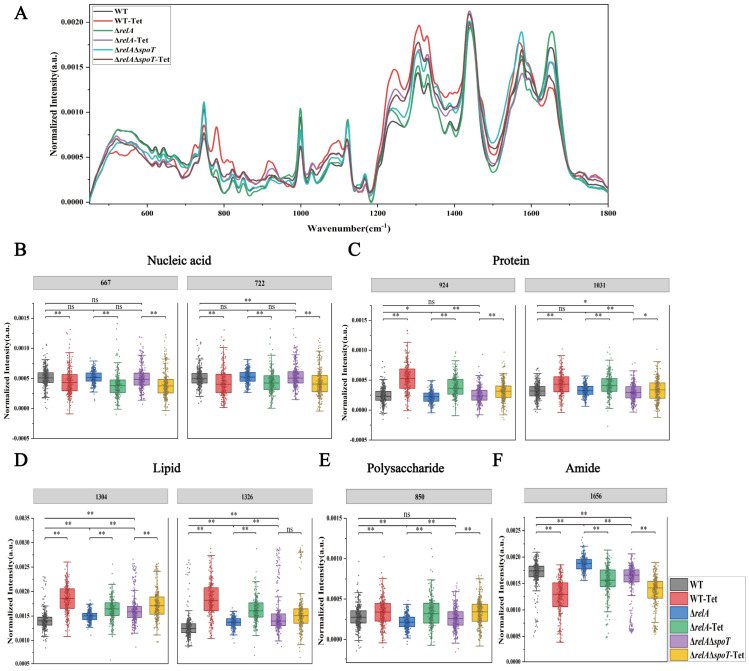
Single-cell Raman spectra of WT, Δ*relA*, Δ*relA*Δ*spoT* and their persisters. (**A**) Mean spectra of *V. splendidus* and its persisters, normalized by the sum of fingerprint area from 450 to 1800 cm^−1^. (**B**–**F**) Intensity of Raman bands for major cellular components: nucleic acid (**B**), protein (**C**), lipid (**D**), polysaccharide (**E**), and amide (**F**). N > 100 for each group. ns indicates no significant difference, * *p* < 0.05, ** *p* < 0.01.

The fingerprint region (450–1800 cm^−1^) of single-cell Raman spectra was further analyzed. By comparing Raman spectra of the WT and mutant strains and their corresponding persisters, significant changes were observed in the intensities of Raman bands corresponding to major intracellular components and metabolites, including nucleic acids, proteins, lipids, polysaccharides and amides (Figure 4). After tetracycline treatment, the intensities of nucleic acid (Figure 4B) and amide (Figure 4F) related bands were reduced in both WT and mutant strains, while the intensities of protein, lipid and polysaccharide related bands were significantly elevated (Figure 4C–E). The difference was that the magnitude of changes in all components was smaller in mutant persisters than in WT persisters. Compared with the untreated WT strains, the untreated Δ*relA* single mutant exhibited increased intensities of lipid and amide bands and decreased polysaccharide band intensity. In contrast, the Δ*relA*Δ*spoT* double mutant showed further enhancement in lipid band intensity, a marked reduction in amide band intensity below the WT baseline, and recovery of polysaccharide band intensity to the WT level. These analytical results indicate that deletion of *relA* and *spoT* significantly affects the biosynthesis and metabolic pathways of *V. splendidus* and its persisters.

### 3.3. Effect of Gene Deletion on Growth and Antibiotic Susceptibility of V. splendidus

**Figure 5:**
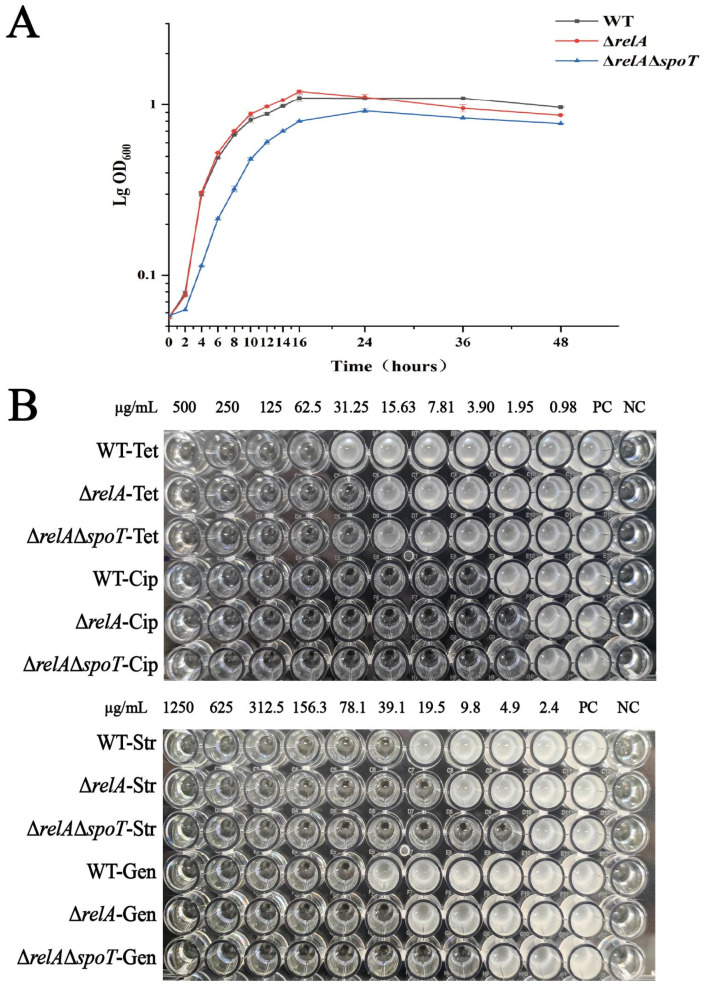
(**A**) Determination of growth curves of WT, Δ*relA* and Δ*relA*Δ*spoT* strains. Error bars represent the standard deviation (n = 3). (**B**) Determination of minimum inhibitory concentration of tetracycline, ciprofloxacin, streptomycin and gentamicin against WT, Δ*relA* and Δ*relA*Δ*spoT* strains. PC represents positive control without antibiotics, and NC represents the negative control without bacterial culture solution. MICs were determined in 2216E medium. Note that persister induction was performed in LB_35_ medium, where the tetracycline MIC for WT is 1.56 μg/mL.

To investigate the effects of *relA* and *spoT* deletion on the growth of *V. splendidus,* the growth curves of the WT, Δ*relA* and Δ*relA*Δ*spoT* were determined. Comparative analysis showed that there was no significant difference in growth rate between the Δ*relA* and WT strains, while the Δ*relA*Δ*spoT* displayed significantly retarded growth relative to WT strain (Figure 5A). These findings indicate that *relA* deletion has no obvious effect on normal bacterial growth, whereas further deletion of *spoT* impairs bacterial growth. Given the dual activity of the SpoT protein, it is speculated that *relA* deletion triggers activation of SpoT synthetase activity, which compensates for RelA function and maintains normal bacterial growth. Subsequent deletion of *spoT* deprives bacteria of intracellular (p)ppGpp synthesis, thereby resulting in growth retardation. These results demonstrate that *relA* and *spoT* are involved in regulating the growth rate of *V. splendidus*.

Given the impaired persister formation of the mutant strains, we further determined the MICs of four common antibiotics against the WT, Δ*relA* and Δ*relA*Δ*spoT* strains. The results showed that the MIC values of tetracycline, ciprofloxacin, streptomycin and gentamicin for both the Δ*relA* and Δ*relA*Δ*spoT* strains were lower than those of the WT. Moreover, regarding aminoglycoside antibiotics (streptomycin and gentamicin), the MIC for the Δ*relA*Δ*spoT* strain were lower compared with the Δ*relA* strain (Figure 5B). These results indicate that deletion of *relA* and *spoT* may affect the antibiotic susceptibility of *V. splendidus*.

### 3.4. Effect of Gene Deletion on Virulence-Related Phenotypes of V. splendidus

**Figure 6:**
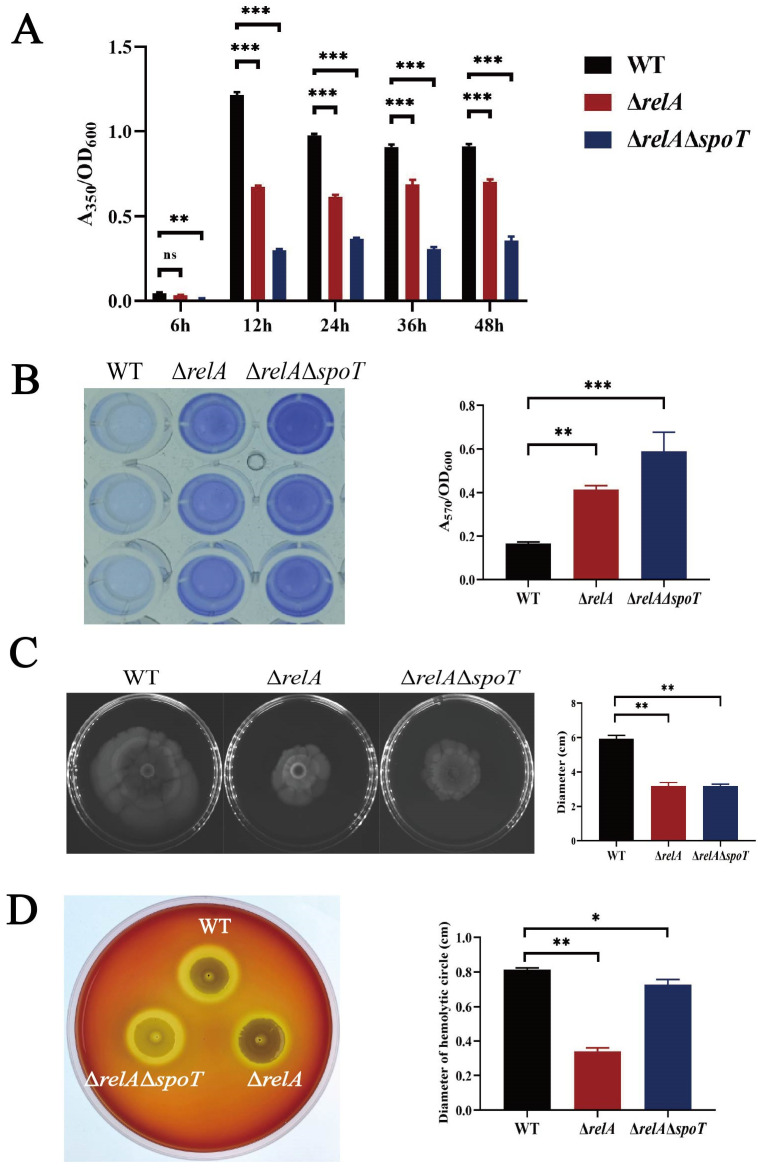
Determination of virulence-related phenotypes of WT, Δ*relA* and Δ*relA*Δ*spoT*. (**A**) Determination of protease activity. (**B**) Determination of biofilm formation using crystal violet. (**C**) Determination of bacterial motility. (**D**) Determination of bacterial hemolytic activity: Error bars represent the standard deviation of three replicate experiments (n = 3). ns indicates no significant difference, * *p* < 0.05, ** *p* < 0.01, *** *p* < 0.001.

The stringent response is not only involved in regulating bacterial persister formation, growth and antibiotic susceptibility, but is also closely associated with bacterial virulence. Firstly, the protease activity of the WT and mutant strains was determined. The results showed that during bacterial culture, protease activity was significantly lower in both Δ*relA* and Δ*relA*Δ*spoT* than in the WT, and the Δ*relA*Δ*spoT* strain displayed further reduced protease activity relative to Δ*relA* (Figure 6A). These findings indicate that *relA* deletion reduces protease production in *V. splendidus*, and subsequent *spoT* deletion leads to an additional decline in protease activity. In addition, biofilm formation capacity was evaluated by crystal violet staining. Compared with the WT, both Δ*relA* and Δ*relA*Δ*spoT* exhibited significantly higher biofilm formation, among which the Δ*relA*Δ*spoT* strain showed the strongest biofilm formation ability (Figure 6B). These results indicate that deletion of *relA* and *spoT* significantly enhances biofilm formation, with a more pronounced promotion effect in the double deletion. This regulatory trend is completely opposite to that of protease activity, suggesting that the stringent response exerts differential regulatory effects on distinct virulence phenotypes. Motility of the WT and mutant strains was assessed using semi-solid agar plates. The WT strain possessed the largest swimming diameter, while the colony diameters of Δ*relA* and Δ*relA*Δ*spoT* were significantly smaller than that of the WT (Figure 6C). Accordingly, deletion of *relA* and *spoT* impairs bacterial motility, suggesting that the stringent response positively regulates motility in this species. Hemolytic activity was evaluated on fresh sheep blood agar plates. After incubation at 28 °C for 48 h, all strains formed hemolytic zones, but with obvious differences in halo size. The WT strain had the largest hemolytic zone. The hemolytic zone diameter of Δ*relA* was significantly smaller than that of the WT, while the Δ*relA*Δ*spoT* strain exhibited partial recovery in hemolytic activity yet remained lower than the WT (Figure 6D). This indicates that *relA* deletion reduces hemolytic activity in *V. splendidus*, and simultaneous deletion of *relA* and *spoT* may activate other compensatory hemolysin expression pathways independent of (p)ppGpp. Overall, diverse virulence traits exhibit distinct response patterns upon the loss of the stringent response, exhibiting characteristics of a complex regulatory network.

### 3.5. Effect of Gene Deletion on Pathogenicity of V. splendidus

**Figure 7:**
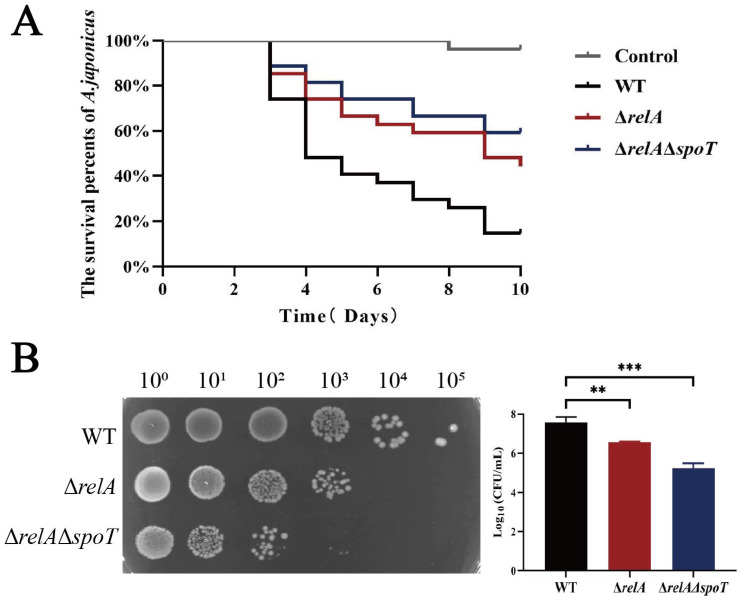
Determination of pathogenicity of WT, Δ*relA* and Δ*relA*Δ*spoT* strains. (**A**) Survival curve of *Apostichopus japonicus* juveniles infected by bacteria. The log-rank (Mantel–Cox) test showed highly significant differences (*p* < 0.001). (**B**) Determination of bacterial invasion ability to coelomocytes of *A. japonicus*: Error bars represent the standard deviation of three replicate experiments (n = 3); ** *p* < 0.01, *** *p* < 0.001.

To elucidate the direct effect of stringent response core gene deletion on the pathogenicity of *V. splendidus* against *A. japonicus*, artificial infection assays were performed. The uninfected *A. japonicus* served as the negative control. *A. japonicus* were challenged with bacterial suspension at a final concentration of 1.0 × 10^7^ CFU/mL. Disease symptoms began to appear in *A. japonicus* at 3 days after infection with *V. splendidus.* Notably, the mortality of *A. japonicus* in mutant-infected groups was significantly lower than that in the WT (Figure 7A). The final survival rate of the WT-infected group was 23%, while the survival rates of the Δ*relA* and Δ*relA*Δ*spoT* groups reached 50% and 63%, respectively. The relative lethality was reduced by 2.2- to 2.7-fold compared with the WT strain. These results were consistent with the virulence-related phenotype assays. The decreased protease activity, hemolytic activity and motility of mutant strains contribute to their reduced pathogenicity.

The invasive ability of the three *V. splendidus* strains against *A. japonicus* coelomocytes was determined using an in vitro co-culture system, and the invasion efficiency was quantified by counting intracellular viable bacteria (CFU/mL). Compared with the WT, both Δ*relA* and Δ*relA*Δ*spoT* exhibited markedly decreased the intracellular bacterial loads after invading coelomocytes. The Δ*relA* strain showed significantly weaker invasion than the WT, and Δ*relA*Δ*spoT* displayed a further reduction in invasive ability (Figure 7B). These results demonstrate that deletion of *relA* and *spoT* significantly attenuates the invasion of *V. splendidus* into *A. japonicus* coelomocytes, with a more severe defect in the double mutant. Thia indicates that the stringent response positively regulates the cellular invasion of *V. splendidus* through *relA* and *spoT*, which serve as a key factor contributing to the reduced pathogenicity of mutant strains.

### 3.6. Transcriptomic Analysis

**Figure 8:**
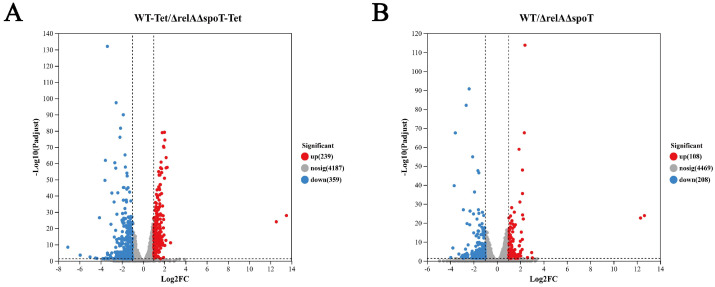
Volcano plot of expression level differences. (**A**) Expression level in WT persisters relative to Δ*relA*Δ*spoT* persisters. (**B**) Expression level in WT relative to Δ*relA*Δ*spoT* strains.

Transcriptome sequencing was conducted on the WT and Δ*relA*Δ*spoT* strains and their persisters to explore the molecular mechanisms by which stringent response regulates persister formation and virulence in *V. splendidus*. Volcano plot analysis of differentially expressed genes showed that 108 genes were significantly upregulated while 208 genes were significantly downregulated in the Δ*relA*Δ*spoT* compared with the WT (Figure 8B). In contrast, 239 upregulated genes and 359 downregulated genes were identified in Δ*relA*Δ*spoT* persisters compared with WT persisters (Figure 8A). These findings indicate that deletion of the stringent response core genes *relA* and *spoT* significantly remodels the transcriptional profiles of *V. splendidus* and its persisters.

**Figure 9:**
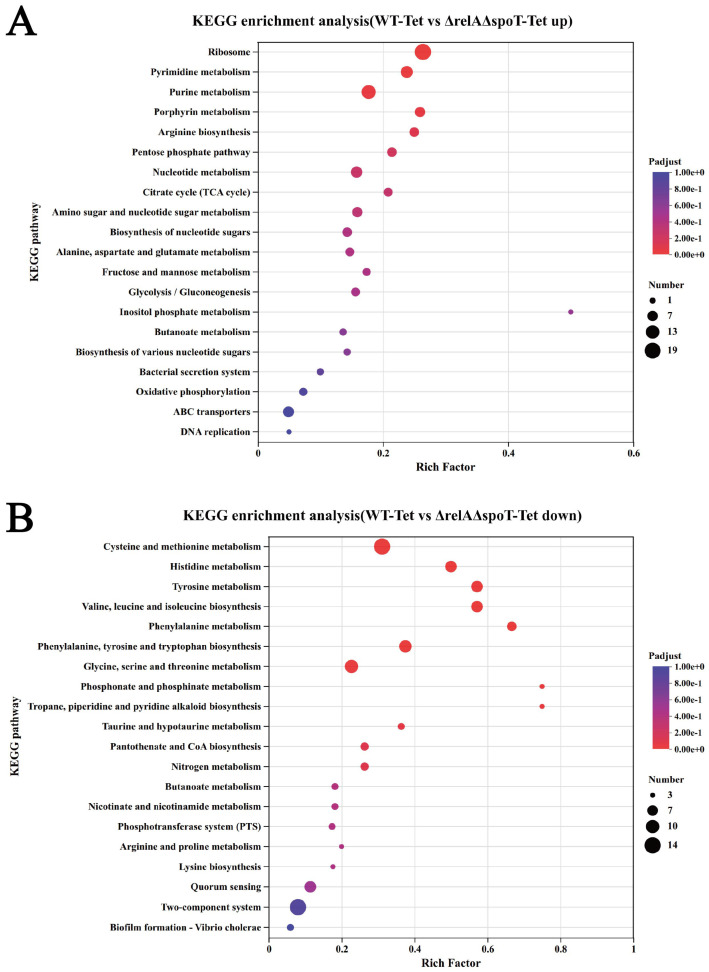
Transcriptome KEGG enrichment analysis. (**A**) Upregulated pathways in WT persisters relative to Δ*relA*Δ*spoT* persisters; (**B**) Downregulated pathways in WT persisters relative to Δ*relA*Δ*spoT* persisters.

KEGG pathway enrichment analysis was conducted on differentially expressed genes, and TPM values of genes were quantified. Multiple metabolic pathways, including ribosome, pyrimidine and purine metabolism, nucleotide metabolism, TCA cycle, glycolysis/gluconeogenesis, and oxidative phosphorylation, were significantly upregulated in WT persisters compared with Δ*relA*Δ*spoT* persisters (Figure 9A). These pathways are essential for persister survival under the regulation of stringent response. WT persisters maintained stable basal metabolism and energy storage capacity, which was consistent with the high metabolic activity revealed by Raman spectroscopy. In contrast, the absence of a stringent response disabled the effective regulation of these core metabolic pathways in mutant persisters. Meanwhile, several pathways were significantly downregulated in WT persisters, such as amino acid biosynthesis, two-component systems, quorum sensing and biofilm formation (Figure 9B). These pathways were highly expressed in the Δ*relA*Δ*spoT* strain and potentially participated in modulating persister formation and survival. This suggested that the double mutant activates alternative regulatory pathways to cope with antibiotic stress after stringent response deficiency. Notably, the sigma factor gene *rpoS* was significantly downregulated in WT persisters compared to Δ*relA*Δ*spoT* persisters (Table 4), indicating that *rpoS* is upregulated in Δ*relA*Δ*spoT* persisters. Sigma factors mediate a series of stress responses, including antioxidant defense, osmoprotection and biofilm formation, which may act as a vital compensatory mechanism to sustain persister formation in the double mutant lacking the stringent response.

**Figure 10:**
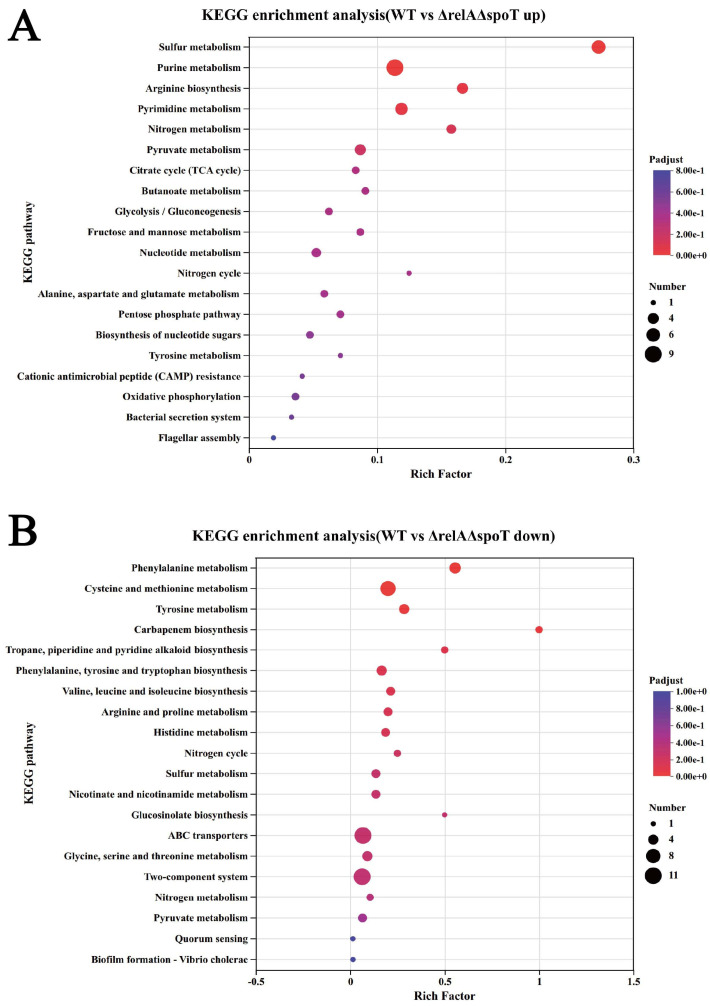
Transcriptome KEGG enrichment analysis. (**A**) Upregulated pathways in WT relative to Δ*relA*Δ*spoT* strains; (**B**) Downregulated pathways in WT relative to Δ*relA*Δ*spoT* strains.

Purine metabolism, pyrimidine metabolism, nucleotide metabolism, nitrogen metabolism, the TCA cycle, and oxidative phosphorylation were significantly upregulated in the WT relative to the Δ*relA*Δ*spoT* strain (Figure 10A). These pathways underpin ATP production, ribosome synthesis, and cell division, reflecting the robust biosynthetic and growth capacity of the WT. Conversely, after losing the regulation of the stringent response, the above pathways were downregulated, exhibiting reduced central carbon metabolism, insufficient energy production and impaired nucleotide synthesis, which is consistent with its growth retardation phenotype. Meanwhile, flagellar assembly, bacterial secretion systems and cationic antimicrobial peptide (CAMP) resistance were significantly upregulated in the WT. This indicated that the stringent response enhanced motility, virulence protein secretion efficiency, and host immune defense resistance in *V. splendidus*. The reduced expression of these pathways in Δ*relA*Δ*spoT* corresponded to its decreased motility, protease activity, pathogenicity and cellular invasion capacity. Moreover, ABC transporters, two-component systems, quorum sensing and *Vibrio cholerae*-type biofilm formation were downregulated in the WT (Table 5; Figure 10B), implying that they are relatively active in Δ*relA*Δ*spoT*. This is consistent with the enhanced biofilm formation of the double mutant, suggesting that stringent response deficiency may promote bacterial group behaviors and biofilm development. In conclusion, the stringent response globally modulates central metabolism, virulence-related pathways and group behavior-related pathways in *V. splendidus*, and plays an important role in bacterial proliferation, virulence regulation and biofilm formation.

## 4. Discussion

In this study, single-knockout (Δ*relA*) and double-knockout (Δ*relA*Δ*spoT*) strains were constructed to investigate the effects of stringent response deficiency on persister formation and biological characteristics of *Vibrio splendidus*. Consistent with previous findings in *E. coli* K-12, impaired (p)ppGpp synthesis reduces persister abundance [9]. Interestingly, a partial recovery in persister counts was observed in the Δ*relA*Δ*spoT* compared with the Δ*relA* strain, although levels remained lower than those in the WT. This may be related to the dual synthetase/hydrolase activity of the SpoT protein. When the stringent response is completely abolished, other alternative persister formation pathways may be activated. However, further verification is required. Nevertheless, persister levels of the knockout strains were significantly lower than those of the WT, confirming that the stringent response acts as a positive regulator of persister formation in *V. splendidus*. This is consistent with observations in other bacterial species [37,38]. Notably, stringent response deficiency did not completely eliminate persisters in *V. splendidus*. Similar findings have been reported in *E. coli* [39], suggesting that persister formation is controlled by multiple combined pathways [40,41].

D_2_O-labeled single-cell Raman spectroscopy provides a powerful tool for investigating metabolic activity and cellular component changes in persisters [42,43]. Multiple studies have reported that antibiotic-mediated growth inhibition does not equal metabolic suppression, and non-growing bacteria may still retain metabolic activity [44,45,46]. This view challenges the traditional concept that deep dormancy dominates the antibiotic tolerance of persisters [47,48]. In the present study, active metabolism was detected after tetracycline treatment in the WT *V. splendidus*, which supports the emerging viewpoint of metabolically active persisters reported in *E. coli* [27] and *M. tuberculosis* [26]. These findings indicate that persisters are not completely dormant but possess active metabolic regulatory capabilities. Compared to the WT persisters, the Δ*relA* persisters had decreased metabolic activity, while Δ*relA*Δ*spoT* persisters showed partial recovery, which is consistent with persister number changes. Raman fingerprint region analysis revealed that the mutant persisters undergo significant molecular remodeling. Both the WT and mutant persisters presented decreased nucleic acid and amide band intensities and elevated protein, lipid and polysaccharide bands; whereas the variation magnitude in the mutant strains was relatively mild. These findings indicate that bacteria employ multiple adaptive strategies to respond to environmental stress, and the stringent response is involved. Notably, the Δ*relA*Δ*spoT* strain showed drastically increased lipid signals and the lowest amide signals, which may represent a vital adaptive survival strategy under complete stringent response deficiency. It has been verified that (p)ppGpp can directly inhibit key enzymes, including acetyl-CoA carboxylase and β-hydroxyacyl-ACP dehydratase [5]. When (p)ppGpp is completely absent, these inhibitory effects are relieved, which may lead to excessive lipid accumulation. The reduced amide signal reflects an overall decline in protein synthesis capacity, which corroborates the growth retardation observed in the Δ*relA*Δ*spoT* strain. Recent studies have shown that the growth of an *E. coli* (p)ppGpp^0^ strain was inhibited [49]. This is mainly due to the lack of (p)ppGpp regulation of ribosome synthesis, which leads to an unnecessary excessive accumulation of ribosomes and hinders the synthesis of required metabolic proteins, thereby affecting their growth capacity.

Results of virulence-associated phenotypic assays further revealed the complexity of the stringent response-mediated regulatory network. Both the Δ*relA* and Δ*relA*Δ*spoT* strains exhibited significantly decreased protease activity. As an important extracellular virulence factor, bacterial proteases facilitate host tissue protein degradation. (p)ppGpp modulates the synthesis and secretion of secreted proteins, and its reduction inhibits protease gene expression and secretion [50]. Moreover, the absence of the stringent response significantly impaired the motility of *V. splendidus*. Similarly, the Δ*relA*Δ*spoT* mutant of *P. aeruginosa* displays inhibited surface motility and swarming motility, which are coregulated by stringent response downstream genes and quorum-sensing systems [51,52]. Consistent with our findings, enhanced biofilm formation was reported in *Actinobacillus pleuropneumoniae* S8 Δ*relA* [53] and *Campylobacter jejuni* Δ*spoT* [54]. However, impaired (p)ppGpp synthesis was shown to prevent structured biofilm formation in *E. coli* and *P. aeruginosa* [51,55], indicating species-specific regulation. The opposite regulation of motility and biofilm formation in the mutants likely reflects an adaptive response, in which enhanced biofilm formation compensates for reduced motility to improve survival. Hemolytic activity presented a distinct non-additive pattern: a decrease in Δ*relA* and a partial recovery in Δ*relA*Δ*spoT*. This non-additive behavior suggests that hemolysin production or secretion is governed by a more intricate regulatory architecture. Despite the enhancement of biofilm formation and the partial recovery of hemolytic activity, stringent response-deficient strains had significantly reduced pathogenicity toward *A. japonicus* and impaired invasion of its coelomocytes. Studies have shown that defects in (p)ppGpp synthesis similarly attenuate host toxicity in *Staphylococcus aureus* [56] and *P. aeruginosa* [51]. Collectively, these findings reinforce the view that the stringent response is a critical determinant of virulence across diverse pathogens, though the specific effector pathways may be more nuanced [5,53].

Transcriptome profiling provides a molecular framework for these phenotypic observations. In the Δ*relA*Δ*spoT* strain, differentially expressed genes were significantly enriched in multiple metabolic pathways, including nucleotide metabolism, amino acid metabolism, central carbon metabolism and ABC transporter pathways. Altered nutrient acquisition, metabolic regulation, and survival strategies in the mutant lead to changes in growth, virulence, and biofilm formation, reflecting an adaptive shift following the loss of the stringent response [57]. In the persister state, Δ*relA*Δ*spoT* exhibited a downregulation of pathways related to the ribosome, energy metabolism, and purine/pyrimidine metabolism compared to WT persisters, indicating that the stringent response is essential for maintaining the basal metabolism of the persister. Notably, the sigma factor gene *rpoS* and pathways associated with two-component systems, quorum sensing, and biofilm formation were relatively upregulated in Δ*relA*Δ*spoT* persisters. The involvement of *rpoS* in persister formation and its functional interaction with (p)ppGpp have been well documented [58]. The activation of these alternative stress-responsive circuits may partially compensate for the loss of stringent response signaling. In conclusion, the stringent response globally regulates the persister formation, growth, and virulence of *V. splendidus* by modulating energy metabolism, nutrient biosynthesis, and multidimensional stress response networks. It should be noted that this study has several limitations. First, we did not directly quantify intracellular (p)ppGpp levels, and such biochemical detection would provide more direct evidence to support our mechanistic interpretations. Second, genetic complementation assays were not conducted for the knockout strains. Thus, we cannot rule out the possibility that the partial phenotypic recovery in the Δ*relA*Δ*spoT* double mutant may result from off-target mutations or adaptive genetic changes during strain construction. Further validation via gene complementation or other independent experiments is therefore required to confirm the proposed compensatory mechanisms.

## Figures and Tables

**Table 1:** Strains and plasmids listed in this study.

Strains/Plasmids	Description	Reference/Source
*Vibrio splendidus* AJ01	Wild-type strain	Lab stock
Δ*relA*	AmpR, derivative of *V. splendidus* AJ01	This study
Δ*relA*Δ*spoT*	AmpR, derivative of *V. splendidus* AJ01	This study
*E. coli* DH5α	Host strain	Takara
*E. coli* S17λπ	Host strain	Takara
pK18mobsacB	KanR, expression vector	Lab stock

**Table 2:** The main information of primers.

Primer Name	Sequence (5′–3′)	Information
KoRelAF1	CAGGTCGACTCTAGAGGATCCTGTTTTGTGGTTTGGGGAACTT	knockout
KoRelAR1	ATCCCAACCTTGTACCGCAACCATCATGCCT	knockout
KoRelAF2	TTGCGGTACAAGGTTGGGATAGTCCAAAATACTATC	knockout
KoRelAR2	CTATGACATGATTACGAATTCTTTCTTCATCACTCGCA	knockout
KoSpoTF1	CAGGTCGACTCTAGAGGATCCCGCCAGCATCTTACGTTTACC	knockout
KoSpoTR1	CGATAAGACGTCGTAAGAACTAGATCCGG	knockout
KoSpoTF2	GTTCTTACGACGTCTTATCGAATAGATACAAATTAAAGGCCC	knockout
KoSpoTR2	CTATGACATGATTACGAATTCTGATGCAGCCTTAATGGACTTCA	knockout
27F	AGAGTTTGATCMTGGCTCAG	strain identification
1492R	GGTTACCTTGTTACGACTT	strain identification

**Table 3:** Molecular assignment of the Raman peaks found in this study.

Wavenumber (cm^−1^)	Molecular Assignment	Components	Reference
667	Guanine, tyrosine-G backbone in RNA	Nucleic acids	[27,28,29,30,31,32,33,34]
722	Adenine, DNA	Nucleic acids	
850	Polysaccharide structure	Polysaccharide	
924	ν(C-C)	Protein	
1031	δ(C-H) bend., Tyr, Phe	Protein	
1304	CH_2_ deformation	Lipid	
1326	C-H deformation, CH_2_	Lipid	
1656	Amide I	Amide	

**Table 4:** Differentially expressed genes in WT persisters relative to Δ*relA*Δ*spoT* persisters.

Pathways	Gene	Gene Description	Regulate	*p*-Value
Ribosome	*rpmH*	50S ribosomal protein L34	up	2.62 × 10^−35^
	*rplA*	50S ribosomal protein L1	up	6.08 × 10^−30^
	*rpsG*	30S ribosomal protein S7	up	3.18 × 10^−46^
Nucleotide metabolism	*adk*	adenylate kinase	up	8.30 × 10^−18^
	*gmk*	guanylate kinase	up	1.14 × 10^−17^
	*upp*	uracil phosphoribosyltransferase	up	2.92 × 10^−9^
Two-component system	*dnaA*	chromosomal replication initiator protein	up	1.11 × 10^−17^
	*cpaB*	Flp pilus assembly protein	down	4.87 × 10^−4^
	*torS*	TMAO reductase system sensor histidine kinase/response regulator	down	1.79 × 10^−3^
Biofilm formation	*rpoS*	RNA polymerase sigma factor	down	1.24 × 10^−93^
	*oxyR*	DNA-binding transcriptional regulator	down	5.02 × 10^−5^
Quorum sensing	*aroG*	3-deoxy-7-phosphoheptulonate synthase	down	3.00 × 10^−85^
	*cqsA*	alpha-hydroxyketone-type quorum-sensing autoinducer synthase	down	2.30 × 10^−3^

**Table 5:** Differentially expressed genes in WT relative to Δ*relA*Δ*spoT*.

Pathways	Gene	Gene Description	Regulate	*p*-Value
Nucleotide metabolism	*deoA*	thymidine phosphorylase	up	2.26 × 10^−20^
	*manA*	mannose-6-phosphate isomerase type I	up	1.80 × 10^−17^
	*xdhA*	xanthine dehydrogenase small subunit	up	2.50 × 10^−9^
Oxidative phosphorylation	*frdA*	fumarate reductase (quinol) flavoprotein subunit	up	2.44 × 10^−25^
	*frdD*	fumarate reductase subunit	up	8.31 × 10^−24^
	*ccoN*	cytochrome C and quinol oxidase polypeptide I	up	8.47 × 10^−4^
Two-component system	*cheY*	response regulator	up	2.96 × 10^−4^
	*mcp*	methyl-accepting chemotaxis protein	down	5.57 × 10^−19^
	*uhpT*	hexose-6-phosphate:phosphate antiporter	down	5.82 × 10^−6^
Flagellar assembly	*flgF*	flagellar basal body rod protein	up	8.52 × 10^−6^
ABC transporters	*malE*	maltose/maltodextrin ABC transporter substrate-binding protein	up	3.50 × 10^−62^
	*lolC*	lipoprotein-releasing ABC transporter permease subunit	down	6.04 × 10^−4^
	*phnD*	phosphate/phosphite/phosphonate ABC transporter substrate-binding protein	down	7.68 × 10^−3^
Quorum sensing	*aroG*	3-deoxy-7-phosphoheptulonate synthase	down	1.34 × 10^−94^

## Data Availability

The original contributions presented in this study are included in the article. Further inquiries can be directed to the corresponding author.

## Abbreviations

The following abbreviations are used in this manuscript:

WT	Unedited wild-type bacteria
Tet	Tetracycline
Amp	Ampicillin
Kan	Kanamycin
Str	Streptomycin
Gen	Gentamicin
Cip	Ciprofloxacin
